# Medicinal Plants, Phytochemicals, and Herbs to Combat Viral Pathogens Including SARS-CoV-2

**DOI:** 10.3390/molecules26061775

**Published:** 2021-03-22

**Authors:** Arumugam Vijaya Anand, Balasubramanian Balamuralikrishnan, Mohandass Kaviya, Kathirvel Bharathi, Aluru Parithathvi, Meyyazhagan Arun, Nachiappan Senthilkumar, Shanmugam Velayuthaprabhu, Muthukrishnan Saradhadevi, Naif Abdullah Al-Dhabi, Mariadhas Valan Arasu, Mohammad Iqbal Yatoo, Ruchi Tiwari, Kuldeep Dhama

**Affiliations:** 1 Medical Genetics and Epigenetics Laboratory, Department of Human Genetics and Molecular Biology, Bharathiar University, Coimbatore 641046, India; kaviyamohandass@gmail.com (M.K.); bharathikathir30@gmail.com (K.B.); parithathvialuru98@gmail.com (A.P.); 2 Department of Food Science and Biotechnology, College of Life Science, Sejong University, Seoul 05006, Korea; bala.m.k@sejong.ac.kr; 3 Department of Life Sciences, CHRIST (Deemed to be University), Bengaluru 560029, India; arun47biotech@gmail.com; 4 Institute of Forest Genetics and Tree Breeding (IFGTB), Forest Campus, Cowley Brown Road, RS Puram, Coimbatore 641002, India; senthilnk@icfre.org; 5 Department of Biotechnology, Bharathiar University, Coimbatore 641046, India; velayuthaprabhu@yahoo.com; 6 Department of Biochemistry, Bharathiar University, Coimbatore 641046, India; saradhadevi@buc.edu.in; 7 Department of Botany and Microbiology, College of Science, King Saud University, P.O. Box 2455, Riyadh 11451, Saudi Arabia; naldhabi@ksu.edu.sa (N.A.A.-D.); mvalanarasu@gmail.com (M.V.A.); 8 Xavier Research Foundation, St. Xavier’s College, Palayamkottai, Thirunelveli 627002, India; 9 Faculty of Veterinary Sciences and Animal Husbandry, Sher-E-Kashmir University of Agricultural Sciences and Technology of Kashmir, Shalimar, Srinagar 190006, India; iqbalyatoo@gmail.com; 10 Department of Veterinary Microbiology and Immunology, College of Veterinary Sciences, UP Deen Dayal Upadhayay Pashu Chikitsa Vigyan Vishwavidyalay Evum Go-Anusandhan Sansthan (DUVASU), Mathura 281001, India; ruchi.vet@gmail.com; 11 Division of Pathology, ICAR-Indian Veterinary Research Institute, Izatnagar, Bareilly 243122, India

**Keywords:** COVID-19, medicinal plants, phytochemicals, herbs, antiviral agents, SARS-CoV-2

## Abstract

The coronavirus disease 2019 (COVID-19) pandemic, caused by severe acute respiratory syndrome corona virus-2 (SARS-CoV-2), is the most important health issue, internationally. With no specific and effective antiviral therapy for COVID-19, new or repurposed antiviral are urgently needed. Phytochemicals pose a ray of hope for human health during this pandemic, and a great deal of research is concentrated on it. Phytochemicals have been used as antiviral agents against several viruses since they could inhibit several viruses via different mechanisms of direct inhibition either at the viral entry point or the replication stages and via immunomodulation potentials. Recent evidence also suggests that some plants and its components have shown promising antiviral properties against SARS-CoV-2. This review summarizes certain phytochemical agents along with their mode of actions and potential antiviral activities against important viral pathogens. A special focus has been given on medicinal plants and their extracts as well as herbs which have shown promising results to combat SARS-CoV-2 infection and can be useful in treating patients with COVID-19 as alternatives for treatment under phytotherapy approaches during this devastating pandemic situation.

## 1. Introduction

The severe acute respiratory syndrome coronavirus-2 (SARS-CoV-2) causing coronavirus disease 2019 (COVID-19) has become a major pandemic, which has rapidly spread to more than 215 countries, causing serious global health concerns, panic, and huge economic losses [1]. The virus has caused nearly 2.6 million deaths, and 117 million are affected as of 10 March 2021. Lack of specific treatment against SARS-CoV-2 has rendered the world helpless hence various countries are exploring phytochemicals obtained from medicinal plants and herbs as alternatives for treating COVID-19 patients via phytotherapy approaches [2,3,4,5,6]. Recent publications on SARS-CoV-2/COVID-19 suggest that phytochemicals used to treat the human immunodeficiency virus (HIV) infection can be explored for COVID-19 treatment [7]. Some of the most promising small plant molecules found to inhibit coronavirus are conjugated with fused ring structures and are classified as polyphenols [8]. In an in silico study conducted with SARS-CoV-2 main protease (M^pro^) and angiotensin-converting enzyme 2 (ACE2) as targets in treating coronavirus infection, it was found that absinthin, quercetin 3-glucuronide-7-glucoside, and quercetin 3-vicianoside have a good binding potential to these targets [9]. Therefore, reliable and detailed knowledge of the structure of SARS-CoV-2 and pathogenesis of COVID-19 and also of plant phytochemicals will help us find a treatment to this coronavirus. SARS-CoV-2 has certain important factors that affect its virulence: the spike proteins that mediate its entry into the host, the nucleocapsid that protects its genetic material, and the RNA through which the virus replicates in the host [10]. There are various plants, including *Glycyrrhiza glabra*, *Azadirachta indica*, *Andrographis paniculata*, *Calotropis gigantea*, *Ocimum sanctum*, *Curcuma longa*, *Withania somnifera*, *Zingiber officinale*, *Allium sativum*, *Tinospora cordifolia*, *Moringa oleifera,* and others, that have anti-viral and immunomodulatory properties [11,12,13]. Plant-specific compounds, such as lignans, saponins, alkaloids, kaempferol, luteolin, apigenin, baicalin, quercetin, catechins, flavonoids, and polysulphates (sulphated polysaccharides) play various roles in inhibiting viral entry, destroying the nucleocapsid and genetic material, and inhibiting the replication of viruses, which includes, dengue, herpes simplex virus (HSV), hepatitis C virus (HCV), influenza, chikungunya, SARS, and others [13]. This review discusses the structure of SARS-CoV-2 and its pathogenesis, which will help understand its mechanism of infection. It presents collective information on various plants and their phytochemicals as well as potent herbs that have already been identified as potent antiviral agents against important human pathogens along with their promising applications to safeguard against SARS-CoV-2 infection and usefulness in treating COVID-19 patients as alternative and complementary phytotherapy approaches.

## 2. Structure and Pathogenesis of SARS-CoV-2

**Figure 1:**
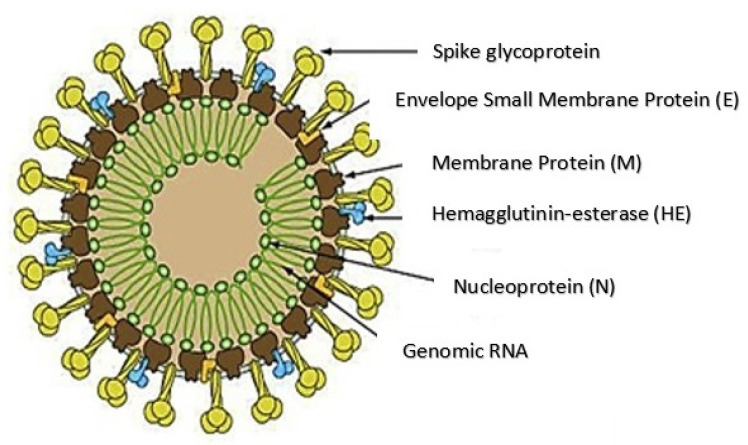
Structure of COVID-19 [Source: http://ruleof6ix.fieldofscience.com/2012/09/a-new-coronavirus-should-youcare html (accessed on 20 June 2020)].

SARS-CoV-2 has a positive-sense, single-stranded RNA that is associated with nucleoproteins present in its capsid comprising matrix proteins [14]. The envelope is made up of club-shaped glycoprotein projections and few coronaviruses have hemagglutinin esterase (HE)-protein [15] in their envelope. SARS-CoV-2 contains four different structural proteins: the spike (S), envelope (E), membrane (M), and nucleocapsid (N) proteins, which is encoded by open reading fragments located on one-third of the genome near the 3’ terminal. Apart from the main structural proteins, it also has other structural and accessory proteins (HE, 3a/b protein, and 4a/b protein) that play various roles in the replication and genome maintenance [16,17]. The membrane glycoprotein (M) is the most common structural protein and it covers the membrane bilayer. It has a short NH_2_-domain situated on the outside and a long -COOH terminal located within the virion [15]. The spike protein plays an important role as an inducer of neutralizing antibodies and also acts as a type I membrane glycoprotein along with peplomers. Figure 1 depicts the structure of SARS-CoV-2. 

**Figure 2:**
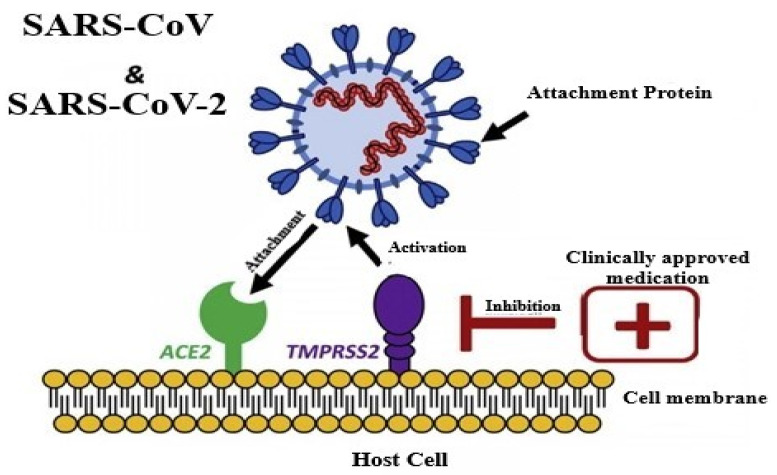
Pathology of COVID-19 [Source: Leila Mousavizadeh, Sorayya Ghasemi, Genotype and phenotype of COVID-19: Their roles in pathogenesis. Journal of Microbiology, Immunology and Infection. 2020].

SARS-CoV-2 enters the host through binding of its spike proteins to ACE2 receptors, and this process is primed with the help of a protease called TMPRSS2 [18,19]. After entry, the virus gets uncoated and starts genome replication and translation at the cytoplasmic membrane with the help of a coordinated process of RNA synthesis (continuous and discontinuous) mediated by a complex of the protein encoded by 20kb replicase gene [20]. The coronaviruses have a replicase enzyme that is not found in other RNA viruses, with the presence of the putative sequence-specific endoribonuclease, 3′-to-5′exoribonucleases, 2′-*o*-ribo methyltransferases, and ADP-ribose-1’-phosphatase [21]^.^ The mechanism of pathogenesis is represented in Figure 2.

## 3. Similarities of Viruses to SARS COV-2

The idea of SARS-CoV-2 structure and pathology will help in comparing viruses that share certain similarities. The zika virus is a single-stranded positive-sense RNA virus with nucleocapsid, the open reading frames codes a single protein which is processed into the capsid, membrane protein, and envelope structural proteins [22]. The rabies virus belongs to the RNA viruses. Although it is a negative RNA virus it has the lipid bilayer membrane covered with transmembrane glycoprotein spikes and a nucleocapsid that covers its genetic material [23]. Dengue virus has a positive-sense RNA [24]. The H1NI (swine flu virus) also affects the respiratory tract with a minimum incubation period of 5 to 7 days and it is an enveloped virus with the glycoprotein spikes on the lipid bilayer membrane and also hemagglutinin on the envelope [25].

The chikungunya virus is also a spherical virus with an envelope consisting of glycoprotein spikes and a positive-sense single-stranded RNA [26]. The Ebola virus even though a tubular-shaped virus with negative-stranded RNA has a lipid bilayer membrane and glycoprotein spikes [27]. SARS-CoV2 M^pro^ and HCV NS3/4A protease shows similarity in three-dimensional structure and also in the arrangement of active site residues. Besides, 8 protease inhibitors of HCV are also capable of binding to M^pro^ active site suggesting that protease inhibitors of HCV can effectively inhibit SARS-CoV-2 protease and the replication of SARS-CoV2 [28]. Spike protein HE found in SARS-CoV2 and hemagglutinin of influenza virus has a similar function [29]. HIV has two copies of single-stranded positive-sense RNA and belonging to the retrovirus family [30]. All the above-mentioned viruses can be compared to their genetic material like RNA viruses or positive-sense RNA virus, a structure like spherical shape, glycoprotein spikes, hemagglutinin, lipid bilayer, nucleocapsid, and the site of infection with SARS CoV-2.

## 4. Plants with Antiviral Properties

Medicinal plants and herbs have shown promising anti-viral properties and multiple beneficial health applications as well as are being used as traditional practitioners to protect various health issues of humans and animals [11,12,13]. Since finding drugs and treatment options for coronaviruses (CoVs), the medicinal plants and their derived phytoconstituents, herbs could provide the strong base for designing and developing the novel alternative and supplementary treatment for coronavirus with exploring phytotherapy approaches. Various medicinal plants extracts, phytochemicals, and herbs have been revised and considered to be the potential anti-CoV agents especially to tackle infection with SARS-CoV-2 for effective control of COVID-19 and future drug development with the medicinal plant formulations for preventing or curing the COVID-19 and other highly infectious viral diseases [2,3,4,5,6,31,32].

A herbal medicine prepared by mixing washed rice water (about 85–90%), endodermis from the roots of *Ulmus pumila* and *Betula luminifera* (about 5–10% and 4–6%, respectively) is used to cure rabies and hydrophobia (one of the symptoms of rabies). This medicine does not show any side effects and is safe for consumption [33]. Pharmaceutical formulations with harmless lectins, for example, with *Sambucus nigra* agglutinin-I, are widely used as antiviral agents for enveloped viruses in animals and humans [34]. The lectins play an important role against the viruses by agglutinating virions and inhibiting them from binding to the cell surface of the host and also by inhibiting the replication of the viruses [11]. Researchers proved that the root extract of *Boerhaavia diffusa* has potential anti-hepatotoxic activity, which can also be used to treat viral hepatitis [35]. Medicinal formulations made by *B. diffusa* alone or in combination with other drugs were found to have antiviral activities against infections associated with the liver, respiratory tract, and heart [36]. The extract of *Eclipta alba* has antiviral activities against many viruses [37]. Its leaf juice is used to cure jaundice and also other ailments of the liver [38]. Besides, the aqueous extract of *Euphorbia prostrate* has antiviral activity against HIV-1 integrase [39].

Medicinal plants inhibit protease enzymes of the SARS-CoV-2 [2]. Many medicinal plants are believed to target the viral 3-chymotrypsin-like cysteine protease (3CL^pro^) enzyme, which is essential for replication of coronavirus [5]. Isoflavone extracted from *Psorothamnus arborescens*, (2*S*)-Eriodictyol 7-*O*-(6″-*O*-galloyl)-beta-d-glucopyranoside from *Phyllanthus emblica*, 3,5,7,3′,4′,5′-hexahydroxy flavanone-3-*O*-beta-d-glucopyranoside from *Phaseolus vulgaris*, methyl rosmarinate from *Hyptis atrorubens*, myricitrin from *Myrica cerifera*, myricetin 3-*O*-beta-d-glucopyranoside from *Camellia sinensis*, amaranthin from *Amaranthus tricolor* and licoleafol from *Glycyrrhiza uralensis* are some of the potent phytochemicals against SARS-CoV-2 [5]. 

Selected quinones are useful in treating HSV, parainfluenza virus, HIV, and *Prunella vulgaris* infections. Some mannose-specific lectins are also used in treating HIV-1 infection. Most of the phytochemicals can be used as reverse transcriptase inhibitors, which are very important for the inhibition of viral infections [40]. Marine-derived lectins are found to possess effective antiviral properties, while plant lectins inhibit viral infections, such as H1N1, H3N2, HIV, and HCV. Lectins obtained from *Galanthus nivalis* are effective in treating HIV1, HIV2, and feline immunodeficiency virus. The diversity of lectins helps treat life-threatening infections, which can lead to epidemics or pandemics [41]. Plants exhibit immunomodulatory characteristics by producing pro-inflammatory cytokines as well as different types of interleukins (IL) secreted by monocytes and dendritic cells, thereby enhancing cell-mediated immunity to fight against viruses [11]. Quinine obtained from the bark of *Cinchona* tree has shown potential as anti-SARS-CoV-2 through its two derivatives *viz*., chlroquine and hydroxychloroquine [42,43,44]. It has been used and is presently being used for treating patients with COVID-19 infection [42,43,44].

Species of the *Veronica* genus are consumed in the form of tonics and applied as ointments to treat influenza and coughs and also used for wound healing, which is known to be inhibiting the intracellular replication of the viruses and symptomatic episodes of HSV-1 infection [45]. Studies carried out in Pakistan conclude that 106 plant species of 56 floral families are effective in treating skin diseases caused by viral infections, such as HIV, also help treat diseases, such as psoriasis, eczema, and leprosy [46]. In vivo studies in mice show that Ayurveda medicines and Chinese folk medicine use drugs and medicines that can cure viral infections, such as those caused by HIV1, HIV2, HSV, influenza virus, Ebola virus, dengue virus, and HCV [47]. Extract from seed coats of the *Caryophyllaceae* family shows antiviral activity against HSV and parainfluenza viruses [48].

## 5. Plants of Indian Origin and Common Use

Numerous plants of important medicinal value in Indian traditional medicine have been quoted to possess anti-SARS-CoV-2 value [3]. *A. indica*, *Ficus religiosa*, *Sesbania grandiflora*, *M. oleifera*, *Avicennia marina*, *Terminalia bellirica*, *P. amarus*, *Hippophae rhamnoides*, are few of these plants having antiviral activity, however, their therapeutic applications in COVID-19 are yet to be investigated [3].

Some spices of Indian origin have also demonstrated anti-SARS-CoV-2 activity by in silico molecular docking approach [49]. They are considered to be effective inhibitors of SARS-CoV-2 M^pro^ enzyme hence having an antiviral effect. However, further validation requires their effectiveness in clinical trials [49]. Common spices needing evaluation are red pepper, garlic, fenugreek, turmeric as they contain active ingredients having diverse medicinal benefits [49]. They may affect proteases [49], RNA binding [50], or envelope protein ion channel of coronaviruses; Gupta et al. [51] and Sinha et al. [52] investigated 20 different active compounds from the *Glycyrrhiza* (liquorice) against spike glycoprotein and non-structural protein-15 endoribonuclease along with lopinavir and rivabirin using the in silico approach. Among the 20 compounds, glyasperin A has a high interaction of nonstructural protein-15 endoribonuclease and glycyrrhizic acid showed the ability to bind spike glycoprotein that inhibited the entry of viruses. Both these compounds were noted to have the high binding ability with the protein receptor cavity by molecular dynamics simulation study, respectively. In vitro and in vivo studies have confirmed that *G. glabra* shows antiviral property against SARS-related coronavirus, H5N1 influenza A virus, HIV-1, HSV, influenza A virus, and respiratory syncytial virus [53]. Glycyrrhizin interferes with oxidative stress induced by H5N1. In lung-derived A549 cells, glycyrrhizin shows inhibition of replication of H5N1 influenza A virus, and also the expression of pro-inflammatory cytokines and apoptosis induced by H5N1 [54].

The ACE-2 favors the entry of SARS-CoV-2 and also supports an anti-inflammatory pathway. Glycyrrhizin and its active metabolite glycyrrhetinic acid have anti-inflammatory activity through Toll-like receptor 4 antagonism, which may reduce the protection of the down-regulated ACE-2. Both are involved in reducing the expression of type 2 transmembrane serine protease, which is crucial for virus uptake [55]. Pan Lau et al. [56] highlighted the therapeutic uses of glycyrrhizin for the remedy of COVID-19 by a mechanism, including the binding with ACE-2, inhibiting thrombin, inhibiting reactive oxygen species, down-regulating pro-inflammatory cytokines, and inducing endogenous interferon (IFN). Glycyrrhizic acid is used to treat viral hepatitis and also have potential activity against other viruses, including SARS-related animal and human coronavirus. Glycyrrhizic acid is an important anti-inflammatory and immuno-active agent that exhibits both membrane and cytoplasmic effects. It makes cholesterol-dependent disorganization of lipid cores that favors the entry of the virus into the host [57]. Nimbidin, nimocinol, nimbolide, nimbinene, isomeldenin, nimbandiol, meliacinanhydride, and zafaral compounds present in *A. indica* leaves have the potential to inhibit COVID-19 M^pro^ [58].

The antiviral capacity of ethanolic and aqueous extracts of *O. sanctum* was examined by infecting Madin-Darby Canine Kidney (MDCK) cells with the H1N1 virus and subsequently treating them with ethanolic and aqueous extracts of *O. sanctum*; the ethanolic extract demonstrated strong antiviral activity against H1N1 at 150 µg/mL [59]. In silico analysis showed that luteolin-7-O-glucuronide and chlorogenic acid present in *O. sanctum* could covalently bind to Cys145 of M^pro^ of SARS-CoV-2 and may hinder the viral enzymes [60].

When HSV-1-infected vero cells were treated with *T. cordifolia*, it inhibited the growth of HSV by 61.43% at 10TCID_50_ [61]. The phytoconstituents in *T. cordifolia*, such asberberine, cardiofolioside B, tinosponone, tembetarine, xanosporic acid have been reported to have a significant docking score. Among these compounds, tinosponone is an important inhibitor of M^pro^ of SARS-CoV-2 and also confirmed the stability of the complex by molecular dynamics simulation [62]. Phytoconstituents present in the *A. sativum* can reduce the expression of pro-inflammatory cytokines, such as leptin and this can play a significant role in prevention of SARS-CoV-2 virus infection [63]. Thuy et al. [64] identified eighteen active substances, which included seventeen organo-sulfur compounds from *A. sativum* [64]. This may interact with the amino acids of the ACE2 protein. Particularly, the allyl di and trisulfide showed strongest anti-coronavirus activity. Moreover, *A. paniculata* showed antiviral activity against influenza *A. flavi* viruses, chikungunya virus, HSV-1, and HIV antigen-positive H9 cells [65]. Murugan et al. [66] investigated the four phytoconstituents, including neoandrographolide, andrographolide, 14-deoxy andrographolide, and 14-deoxy 11,12-didehydro andrographolide from *A. paniculata* by targeting three non-structural proteins, papain-like proteinase and RNA-directed RNA polymerase and also with the structural protein [66]. The results of free energy suggest that neoandrographolide possesses high affinity against SARS-CoV-2 infection. Andrographolide has been noted to inhibit M^pro^ of SARS-CoV-2 by docking analysis [67].

The hydro-alcoholic *W. somnifera* root extract displayed a maximum of 99.9% inhibition of bursal disease virus in chicken embryo fibroblasts at 25 µg/mL in a cytopathic effect reduction assay [68,69,70,71]. Withanolides are natural constituents present in the *W. somnifera* and have been used to treat various diseases traditionally. Tripathi et al. [72] evaluated 40 phytoconstituents from *W. somnifera*. In silico approach revealed that four compounds, such as withanoside II, IV, V, and sitoinodoside IX revealed highest docking energy [72]. Further, withanoside V shows hydrogen-bonding with the active site of the protein and binding affinity. Quercetin glucoside and withanoside X also favor the interactions at the binding site of non-structural protein-15 endoribonuclease and receptor-binding domain [73].

*C. longa* exhibited a decrease in the percentage of cell viability at higher concentrations, and reduction in viral load was observed after 24 h in mice infected with dengue virus [74]. The antiviral activities of curcumin, gallium-curcumin, and Cu-curcumin were tested on HSV-1 infected Vero cell line. The cytotoxic concentration (CC_50_) values for curcumin, gallium-curcumin, and Cu-curcumin were 484.2 µg/mL, 255.8 µg/mL, and 326.6 µg/mL, respectively, with inhibition concentration (IC_50_) values of 33.0 µg/mL, 13.9 µg/mL, and 23.1 µg/mL, respectively. From the results, it has been suggested that curcumin and its derivatives have antiviral activity against HSV-1 [75]. Gupta et al. [76] screened 267 compounds in *C. longa* by docking study [76]. The compounds C1 (1E,6E)-1,2,6,7-tetrahydroxy-1,7-bis(4-hydroxy-3-methoxyphenyl)hepta-1,6-diene-3,5-dione) and C2 (4*Z*,6*E*)-1,5-dihydroxy-1,7-bis(4-hydroxyphenyl)hepta-4,6-dien-3-one were found to be lead agents. Both compounds have a minimum binding score against M^pro^ protein when compared to lopinavir and shikonin and also efficiently bind to the catalytic part of the M^pro^ protein with higher efficacy.

**Figure 3:**
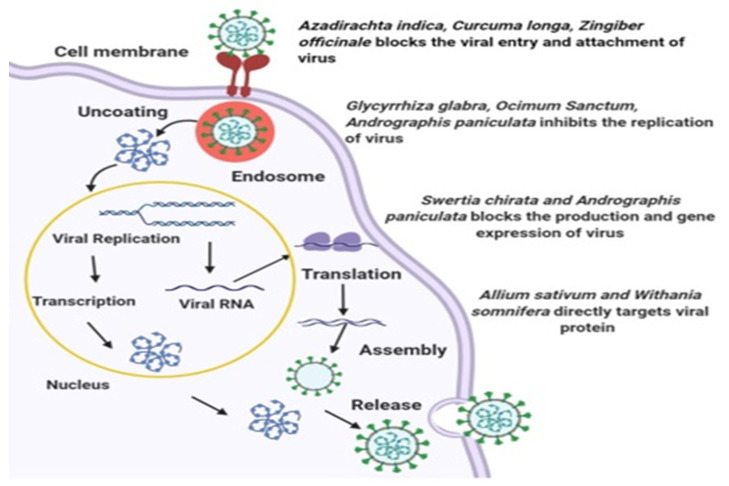
Possible antiviral mechanism of Indian medicinal plants.

Polyphenols of green tea [77] and withanolides of *W. somnifera* [72] are considered to be M^pro^ inhibitors of SARS-CoV-2. Ginseng has proven antiviral, immunomodulatory, anti-inflammatory and antioxidant activity [73]. Ethanolic and aqueous leaf extracts of *M. oleiferai* have kaempferol and anthraquione. Molecular peptide docking of these compounds in comparison with hydroxychloroquine was done and both the compounds revealed important effects regarding the binding of peptides of SARS-CoV-2 [78]. An aqueous extract of the *Phyllanthus* species showed antiviral properties against HSV-1 and HSV-2 [38]. It was also found that *Phyllanthus urinaria* and *P. amarus* possessed significant antiviral activity against HSV-1 and HSV-2 [79]. In a study conducted in Nigeria, leaf extracts of *Macaranga barteri*, *Ipomoea asarifolia, Mondia whitei*, and *Ageratum conyzoides*, as well as *Terminalia ivorensis* bark, showed high antiviral activities against echoviruses [59]. Some Indian plants possessing antiviral properties are tabulated in Table 1 and their possible mechanisms are represented in Figure 3.

Polysulphides are sulphates attached to a carbohydrate backbone or any other polymer. Sulphated polysaccharides have antiviral activity against viruses, especially against some enveloped viruses such as HSV in vitro [85,86], human respiratory syncytial virus, cytomegalovirus, DENV-2, DENV-3, influenza A and B virus, and human hepatoma HepG2 virus. The polysulphates protect against HIV by shielding the CD^4+^ cells against the viral envelope glycoprotein (gp120) at its positively charged V3 loop, that is essential for the attachment of the virus to the primary binding site called the surface heparan sulphate before specific binding occurs through CD^4^ receptors [87]. This mechanism explains its antiviral activity against enveloped viruses. Therefore, it can be speculated that polysulphates might be beneficial in the case of SARS-CoV-2, which is also an enveloped virus, after performing proper investigations [85].

## 6. Plant-Specific Compounds and Antiviral Mechanisms

### 6.1. Flavonoids

Flavonoids are known for their antiviral activity. Many flavonoid compounds are well-known to act as antiviral agents by inhibiting binding and entry of viral, its replication, translation of the viral protein, the formation of envelopes using glycoproteins complexes, and virus release [88]. Flavonoids help in the signaling process in the host cell by activating gene transcription factors and also by secreting cytokines [89]. The structure–activity relationship of flavonoids shows that it is a good inhibitor of the neuraminidase enzyme of influenza virus, thereby preventing its replication [90]. Flavonoids have shown potential in therapy against COVID-19 [91]. They may inhibit SARS-CoV-2 entry into the cell [92] hence have been used in the therapy of COVID-19 patients [93].

### 6.2. Catechins

Green tea contains important catechins (polyphenols), which are of different types, such as (─)-epigallocatecheingallate (EGCG), (─)-epicatechingallate (ECG), and (─)-epogallocatechin (EGC), and has high medicinal values with health benefits [94]. In a quantitative study performed using RT-PCR, high concentrations of EGCG and ECG, but not EGC, decreased viral RNA synthesis in MDCK cells [95]. ECG and EGCG affected the activity of neuraminidase by inhibiting it more efficiently than EGC [95]. The neuraminidase enzyme in viruses is important in transporting budding viruses to other cells by cleaving the sialic acid present in glycoproteins located in the envelope. Similarly, EGCG inhibits both HSV-1 and HSV-2 by binding to their envelope proteins such as gB, gD, or other envelope proteins, which help for the fusion of the virus to cells [96]. Catechin binds the receptor-binding domain of viral S-protein, as well as ACE2 of the host, thus may serve as a therapeutic agent for COVID-19 [97]. In one of the docking analysis study, compound EGCG found in green tea revealed the highest binding affinity with S protein of SARS-CoV-2, which reflects its potential usage in preventing or treating the COVID-19 patients.

### 6.3. Quercetin

Quercetin is a flavonoid compound present in vegetables and fruits [98]. It is found to acts against the HCV virus by inhibiting the action of a heat shock protein that is involved in non-structural protein 5A-mediated translation of viral internal ribosome entry site, which usually occurs in response to stress [99]. Quercetin acts against HCV through the inhibition of HCV NS3 protease, which stops the replication of HCV in the sub genomic RNA replicon cell system [100]. Quercetin halts rhinovirus pathogenesis at different stages of the life cycle of the virus, including endocytosis, protein synthesis, and viral genome transcription [101]. Furthermore, quercetin, along with myricetin, quercetagetin, and baicalin, affected the growth of the Rauscher murine leukemia virus RLV [102]. Quercetin along with vitamin C has been proposed to have the synergistic effect in treating COVID-19 patients [93]. Synergistic antiviral, antioxidant, and immunomodulatory activities and the ability of ascorbate to recycle quercetin, increase the effectiveness of quercetin against SARS-CoV-2 [93].

### 6.4. Apigenin and Baicalin

Apigenin acts against the African swine fever virus by decreasing protein synthesis, thereby causing a three-log decrease in the yield of viruses and is also effective against DNA viruses such as adenoviruses and (hepatitis B virus) HBV [103]. It shows potent antiviral effects against RNA viruses such as picornavirus and acts by inhibiting viral IRES activity, thereby inhibiting the synthesis of viral proteins [104,105]. The translation of enterovirus-71 is disrupted by inhibition of the association of viral RNA with transacting factors that regulate enterovirus-71 [106]. Apigenin is found to disturb HCV virus replication by decreasing the microRNA122, which is a liver-specific microRNA [107].

Baicalin acts against HBV by disrupting its DNA and viral protein synthesis [108]. In H5N1 virus infection, baicalin lowers the levels of interleukin-6 and -8 (IL-6; IL-8) produced but does not interfere with IP-10 levels [109]. Baicalin can inhibit the synthesis of human cytomegalovirus DNA and proteins; however, it does not affect the viral polymerase activity. Baicalin, by interfering with neuraminidase activity, stops the replication of H5N1 in the human lung- and monocyte-derived macrophages [109]. H1N1-infected BALB/c mice administered baicalin orally showed decreased lung virus titers and an increased mean time of death [110]. The results were also found in mice infected with Sendai virus [111]. Studies have shown that baicalin could help in the production of IFN-γ by CD^4+^ and CD^8+^ T cells during infection with influenza virus [112]. In silico studies on baicalin strongly suggest that it has a good binding ability with the NS3/NS2B protein of dengue virus; however, baicalin shows better interactions with NS5 protein.

### 6.5. Luteolin

Luteolin and luteolin-rich fractions are found to have antiviral property, including SARS–CoV, chikungunya virus, Japanese encephalitis virus, and rhesus rotaviruses [113,114,115,116]. Luteolin inhibited HIV-1 by preventing clade B- and C- Tat-driven long terminal repeat (LTR) trans activation [117]. In the case of Epstein-Barr virus, luteolin deregulated the binding of transcription factor Sp1, which decreased the activity of early genes *Zta* and *Rta* [118]. Above all, it was found to be the most potent compound among 400 natural compounds against enterovirus-71 and coxsackievirus A 16 infections, since it disrupts viral RNA replication [119]. Luteolin has antiviral, anti-inflammatory, neurotrophic actions, anti-oxidant, anti-cancer, and anti-apoptotic activities [120]. It has shown the ability to inhibit the entry of SARS-CoV virus and fusion with human receptors, thus may have potential anti-SARS-CoV-2 activity [120].

### 6.6. Kaempferol

The compound kaempferol obtained from *Ficus benjamina* has demonstrated to have a protective effect on HSV-1 and HSV-2, except forits aglycone form [121]. A rhamnose residue containing kaempferol inhibits coronavirus release by affecting 3a channels [122]. Kaempferol and kaempferol-7-*o*-glucoside display inhibitory effect on HIV 1 reverse transcriptase. Besides, kaempferol 3,7-bisrhamnoside isolated from *Taxillus sutchuenensis*, is effective against HCV NS3 protease function [123]. In the case of H1N1 and H9N2 influenza viruses, kaempferol affects neuraminidase activity using specific functional groups [124]. RNA frame shift site (fs RNA) is found to be the target site of kaempferol, which serves to inhibit the Japanese encephalitis virus [125]. Kaempferol has more binding stability and its structural features have shown that it affects binding at the site of N3 in the SARS-CoV-2 M^pro^ [126].

### 6.7. Alkaloids

Lycorine is the most important alkaloids found in the *Amaryllidaceae* family. The lycorine was found to inhibit the poliomyelitis virus in Vero cells at a low concentration of 1 µg/mL but was cytotoxic at a concentration of 25 µg/mL [127]. Lycorine obtained from *Lycoris radiata* had significant antiviral activity against two strains (BJ001, BJ006) of SARS-CoV grown on Vero cells, with an EC_50_ at 15.7 ± 1.2 nM, CC_50_ at 14,980.0 ± 912.0 nM, and a selective index (SI), which is greater than 900 [128]. This SI index is a ratio between the antiviral effect and the toxicity of a compound; the greater the SI value, the safer the drug could be when administered in vitro [129]. Another compound, sophoridine, was found to have antiviral activity against the Enterovirus-71, when Vero cells were pretreated with sophoridine before being infected with this virus [130]. In a study conducted with coxsackievirus in mice, sophoridine obtained from *Sophora flavescens* had a potential role in enhancing the expression of IFN-γ and interleukin-10 (IL-10) to increase the host resistance response against the virus [131]. Among the ipecac, alkaloidsemetine, ipecac alkaloids and analogues are possible antiviral agents for CoVs, hence having prospects for use in COVID-19 therapy [132].

### 6.8. Saponins

Saponin isolated from *Anagallis arvensis* was found to have antiviral property against poliovirus-2 and HSV-1 by protecting the host cells from structural damage [133]. Tormentic acid glucosyl ester, a triterpenoid saponin demonstrated antiviral property against HSV-1 by inhibiting its viral capsid protein synthesis and DNA replication, respectively [134]. Administration of polyphylla saponin I (obtained from *Paris polyphylla*, 5–10 mg/kg) and oseltamivir (3 mg/kg) to mice infected with influenza virus decreased viral hemagglutination titers and reduced pathological conditions in lung tissues of the infected mice, thereby reducing their mortality [135]. Saponins may inhibit the cellular attachment, entry, adsorption, and penetration of a virion into the host cell. Saponins possess immunomodulatory, anti-inflammatory activities, anti-proliferative effect, and antiviral activities including SARS-CoV [4,136,137], hence may have a role in curing COVID-19 patients [136].

### 6.9. Lignans

Lignans are phenolic compounds derived from the shikimic acid biosynthetic pathway in plants [138]. Niranthin obtained from *P. niruriacts* acts against the HBV virus by inhibiting its antigen expression in vitro; it also inhibits duck HBV by inhibiting its DNA replication [139,140]. Nordihydroguaiaretic acid, found in the leaves of *Larrea tridentata*, shows antiviral properties against various viruses, including HCV, dengue virus, influenza A virus, and zika virus by inhibiting genome replication and viral assembly. It affects HCV proliferation by altering host lipid metabolism, interfering with the lipid metabolism and it also suppresses the replication of influenza A virus [140,141,142,143,144]. Terameprocol, semisynthetic compound from lignin, which is derived from the leaves of *L. tridentata* acts against the West Nile virus by affecting viral replication against poxvirus by inhibiting the cell-to-cell transfer of the virus, and against HSV and HIV by preventing viral replication through inhibition of the binding with host transcription factor [145,146,147,148,149]. Arctigenin demonstrates antiviral properties including influenza A virus and HIV-1 by inducing the release of IFNs and also by inhibiting the expression of the proteins (p17 and p24) of the HIV-1 virus [150,151,152,153,154].

The addition of yatein, a compound obtained from the dried leaves of *Chamaecyparis obtusa,* to HeLa cells inhibited the expression of HSV-1ICP0 and ICP4 that arrests DNA synthesis in HSV [155,156]. The compound diphyllin obtained from epigeal parts of the genus *Haplophyllum* inhibits the vacuolar ATPase in zika virus infection; it also interferes with the downstream replication of influenza A virus to inhibit its infection [157,158,159,160,161]. Patentiflorin obtained from the leaves and stem of *Justicia gendarussa* acts against zika virus by impeding its fusion with the host cellular membrane, thus preventing infection by avoiding the acidification of lysosomal or endosomal cells of the target. This acts against HIV-1 by inhibiting its reverse transcriptase enzyme [160,161,162,163,164]. Clemastanin B affects viral endocytosis and ribonucleoprotein export from the nucleus while acting against influenza A virus [164,165,166,167]. Silymarin obtained from the seeds of *Silybum marianum* inhibits HCV production by increasing the expression of anti-inflammatory and anti-proliferative genes, but it does not affect serum albumin levels [168,169]. Thus, having considerable antiviral effects through the inhibition of viral replication, lipid metabolism, apoptosis, protein, and cytokine expression; lignins may have potent anti-SARS-CoV-2 actions as they have shown effects against SARS-CoV also [170,171].

### 6.10. Tannins 

Tannins have potential in targeting viral replication at different stages like attacking their attachment, host replication process, viral particle assembly, and protein transport [172]. Ellagitannins, 1,3,4,6-tetra-O-galloyl-β-d-glucose and geraniin present in *P. urinaria* were found to be useful in suppressing the HSV-1 and HSV-2 respectively [173]. Corilagin and geraniin (ellagitannins) found in *Phyllanthus amarus* reduced the interaction of HIV and its replication [174]. Punicalagin and chebulagic acid, two hydrolysable tannins present in *Terminalia chebula* have been successful in inhibiting the viral entry and transport of virus in HSV-1 [175]. 

According to the study conducted with the combination of ellagitannins like castalagin, vescalagin, and grandinin with acyclovir, the effect of castalagin and vescalagin versus HSV-1 was found to be identical to acyclovir, which interpreted that the combination of ellagitannins with acyclovir was efficient [176,177]. Castalagin followed by vescalagin have highest activity against alphaherpevirus-1 [178]. Castalagin was also found to inhibit the HSV-1 replication with its highest sensitivity being recorded at 0–3 h post viral inoculation [179]. 

**Figure 4:**
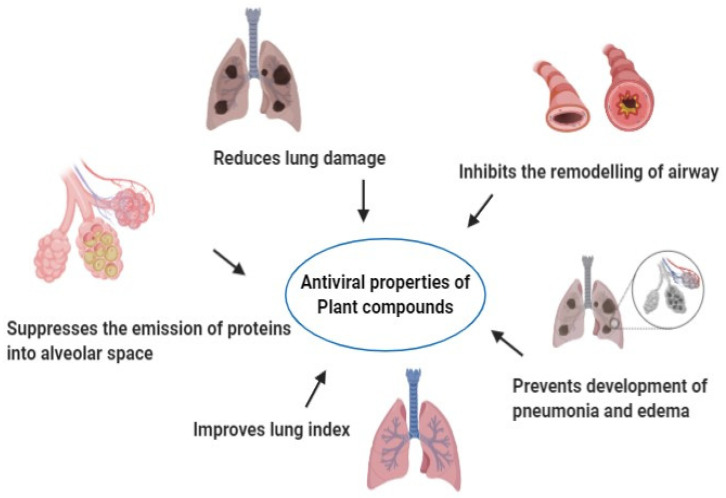
Antiviral properties of plant compounds.

Various plants and herbs have shown effective antiviral and immune-boosting potentials against emerging viruses such as SARS-CoV, zika, ebola, nipah virus, and other highly pathogenic viruses [8,128,166,180,181,182,183]. Apart from developing effective vaccines, therapeutics, and antiviral drugs, the potent antiviral applications of various plants, plant extracts and herbs are required to be endorsed and proliferated optimally by strengthening researches and development activities along with conducting appropriate clinical trials and validation experiments to combat COVID-19 pandemic and its high challenges posed [12,32,184,185,186,187,188,189,190,191]. Advances in the fields of biotechnology, immunology, biochemistry, pharmacology, pharmaceuticals, and nanotechnology may be warranted to their full potential for developing successful antiviral drugs and medicines out of these safe and valuable natural resources against SARS-CoV-2 [192,193,194,195,196,197,198,199,200]. Beneficial applications of medicinal values of plants and herbs could lessen the high incidences, devastating scenario, and public health concerns of SARS-CoV-2/COVID-19. A summary of plant compounds and their antiviral properties is presented in Table 2 while an overview on modes of antiviral action of various phytochemicals/compounds and its derivatives are presented in Table 3 and the Figure 4 represented the antiviral properties of the plant compounds.

## 7. Conclusions

This review presents detailed information about plants and herbs that are widely used to treat viral infections and their phytochemicals that possess antiviral properties. The mechanisms by which the phytochemicals act against the viruses are also elaborated in the review for better understanding. Future studies employing in vitro pharmacological tools to establish the structures of the compounds that inhibit SARS-CoV-2 infection will help in finding a cure for diseases that trigger fast-spreading pandemics. In-depth studies for in vitro and in vivo evaluation of these medicinal plants and their phytochemicals are warranted for assessing anti-SARS-CoV-2 activities. Exploiting the various modes of action of phytoconstituents would lead to practical utilization of the natural resources of plants and herbs for combating this pandemic virus effectively by designing and developing potent drugs and medicines.

## Figures and Tables

**Table 1:** Indian plants with antiviral properties.

Common Name	Botanical and Family Name	Native	Parts Used	Traditional Uses	Antiviral Property
Liquorice or Yashtimadu	*G. glabra* (*Fabaceae*)	Central and Southern Asia, Russia, Northern India (Sub-Himalayan and Punjab), Mediterranean, Afghanistan, and Iran	Roots	Extensively used in Indian traditional medicine systems like Ayurveda and Siddha for ulcer, aliment, purgative, demulcent, antitussive, and expectorant	SARS-related coronavirus, H5N1 influenza A virus, HCV, HIV-1. influenza A virus pneumonia, respiratory syncytial virus and SARS- CoV-2 [53,55]
Neem	*A. indica* (*Meliaceae*)	India, Bangladesh, Burma, Nepal, and West Africa	Leaves, roots, twigs and seeds	Different parts of neem are used as an important ingredient in Ayurveda, Unani and Homeopathy medicine	Dengue virus and SARS-CoV-2 [80,81]
Green chireta	*A. paniculata* (*Acanthaceae*)	South India, Sri Lanka, Pakistan, USA, Thailand, Jamaica, and West Indies	Leaves and roots	The plant has a pivotal role in Chinese and Indian (Siddha and Ayurveda) traditional system for different formulation against various diseases diabetes, sore throat, fever, cirrhosis, malaria, viral hepatitis, liver cancer, and upper respiratory infections	Chikungunya virus, Influenza A, Flaviviruses, HIV antigen-positive H9 cells, and SARS-CoV-2 [65,66]
Tulsi	*O. Sanctum* (*Lamiaceae*)	India, Iran, Italy, Egypt, the USA, and France	Whole plant seeds, leaves and roots	The plant has been well documented in Ayurveda, Siddha, and Greek medicinal system which is used for various treatment purposes such as fever, common cold, malaria fever, epilepsy, bronchitis, migraine, headache, convulsions, hepatic disease, stomach disorders, and heart diseases	H1N1 and SARS-CoV-2 [59,60]
Turmeric	*C. longa* (*Zingiberaceae*)	India, Nepal, China, Bangladesh, and Pakistan	Rhizomes	In Ayurveda, turmeric has a long history of use because of the presence of various beneficial properties used in the treatment of diabetic wounds, fungal infection, cough, rheumatism, hepatic and biliary disorder	Dengue virus, HSV-1 and SARS-CoV-2 [31,74]
Ashwagandha	*W. somnifera* (*Solanaceae*)	India, Sind, Baluchistan, Afghanistan, and Sri Lanka	Roots	The plant is well formulated in Ayurveda, Siddha, Unani and Tibetan Medicine system. Traditionally, *W. somnifera* has been used to treat tumor, stress, immunomodulatory, depression, inflammatory, adaptogenic, and nervous disorder. It is also used in patients with behavioural disturbances for mood stabilization	HSV-1 and SARS-CoV-2 [68,69,70,71]
Garlic	*A. sativum* (*Alliaceae*)	Central Asia, China, Mediterranean region, Mexico, Egypt and in Southern and Central Europe	Cloves, flowers and leaves	Garlic has been traditionally used as hypolipidemic, antihypertensive and anti-thrombotic agent in Ayurvedic, Chinese, and Islamic medicine	Influenza virus A and SARS-CoV-2 [63,64,82]
Guduchi	*T. cordifolia* (*Menispermaceae*)	Indian subcontinent and China	Roots, stem and leaves	The plant is a common shrub used as anti-allergic, anti-inflammatory, antiperiodic, anti-diabetic,, and anti-spasmodic properties in Ayurvedic medicine	HSV-1 and SARS-CoV-2 [62,83]
Drumstick	*M. oleifera* (*Moringaceae*)	Sub-Himalayan tracts of India, Bangladesh, Pakistan, and Afghanistan	Roots, flowers, leaves and pod	The traditional use of plant includes antispasmodic, antiparalytic, antiviral, analgesic, anti-inflammatory, antiepileptic, stimulant and cardiac circulatory tonic	HSV-1 and SARS-CoV-2 [78,84]

**Table 2:** Plant compounds and their antiviral properties.

S. No	Name of the Compound	Structure	Antiviral Property against	Reference
1.	1. FLAVONOIDS	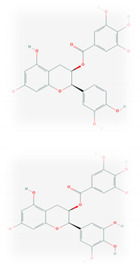	HSV-1 and HSV-2, SARS-CoV-2	[95,97,201]
	1.1. Catechins (Green tea) EGCG and ECG			
	1.2. Quercetin (*C. longa*)	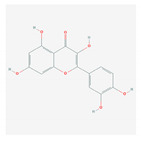	HCV and SARS-CoV-2	[93,99,100]
	1.3. Apigenin (Green tea)	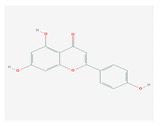	Enterovirus-71, foot and mouth disease virus, HCV, African swine fever virus, and influenza A	[103,104,105]
	1.4. Baicalin (*Scutellaria lateriflora*)	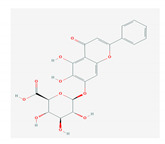	Enterovirus, dengue virus, respiratory syncytical virus, Newcastle disease virus, HIV, and HBV	[108,110,112,120,202,203]
	1.5. Luteolin (*O. sanctum*)	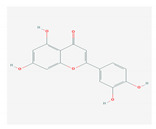	SARS-CoV-2, rhesus rota virus, chickenkuniya virus, and Japanese encephalitis virus	[113,114,115,116,120,204]
	1.6. Kaempferol (*F. benjamina*)	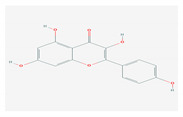	HSV-1, HSV-2, HIV, HCV, H1NI, H9N2, Japanese encephalitis virus, and SARS-CoV-2	[121,123,124,126]
2.	2. ALKALOIDS	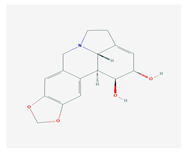	Poliomyelitis virus, SARS-CoV (BJ001 and BJ006)	[127,128,132,205]
	2.1. Lycorine (*L. radiata)*			
	2.2. Sophoridine (*S. flavescens)*	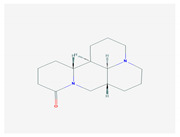	Enterovirus-71 and coxsackievirus	[130,131,206]
3.	3. SAPONINS (*A. arvensis)*	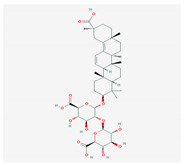	HSV-1, poliovirus, and SARS-CoV 2	[4,134,135,136,207]
4.	4. LIGNANS	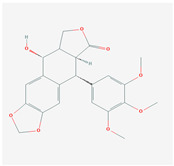	HBV and duck HBV	[139,140,170,171]
	4.1. Nordihydroguairetic acid (*P. niruri.* L)	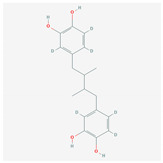	DENV, zika virus or West Nile virus, and influenza A virus	[140,141,142,143,144,208]
	4.2 Arctigenin (*Arctium lappa*)	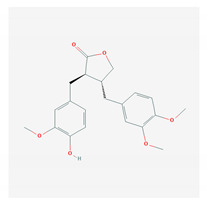	Influenza A virus and HIV-1	[150,151,152,153,154,209]
	4.3. Yatein (*Chamaecyparis obtuse*)	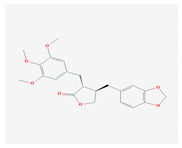	HSV-1	[155,156]
	4.4. Diphyllin (Genus-*Haplophyllum*)	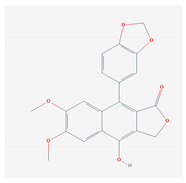	Zika virus and influenza A virus	[157,158,159,160,161,210]
	4.5.Patentiflorin A (*J. gendarussa*)	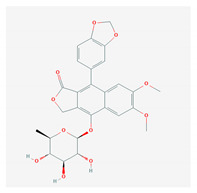	Zika virus and HIV	[160,161,162,163,164,211]
	4.6. Clemastanin B (*Isatis indigotica*)	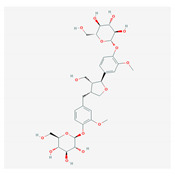	Influenza A virus	[164,165,166,167]
	4.7. Silymarin C (*S. marianum*)	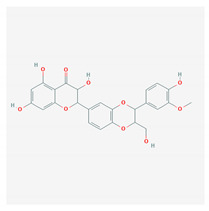	HCV	[168,169,212]
5.	5. TANNINS	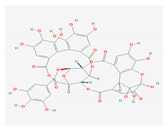	HSV and HIV	[172,173,174,175]
	5.1. Geraniin (*P. amarus)*			
	5.2. 1,3,4,6-tetra-O-galloyl-β-d-glucose (*P. urinaria)*	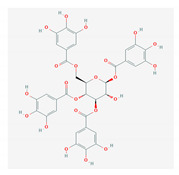		
	5.3. Corilagin (*P. amarus)*	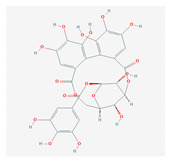		

**Table 3:** Mechanism of antiviral action of phytochemical compounds and its derivatives.

S. No	Name of the Compound	Mechanism of Action	Reference
1.	Polysulphates (sulphated polysaccharides)	Shields the viral envelope gp120 that is essential for the viral attachment	[85,86,87,213]
2.	EGCG and ECG	Decreases the viral attachment in MDCK cells EGCG inhibits the neuraminidase activity more efficiently than EGC EGCG binds to the envelope protein Gb, Gd or other envelope proteins of HSV-1 and HSV-2 that are essential for its fusion with the host cell membrane Catechin binds to the ACE2 and receptor binding domain of viral S-protein of SARS-CoV-2	[95,97,201]
3.	Quercetin	In HCV the heat shock protein activity is inhibited that is essential for non-structural protein 5A mediated viral ribosome entry site and it also inhibits NS3 protease involved in HCV replication Stops the rhinovirus pathogenesis at various steps like endocytosis, protein synthesis and viral genome transcription Vitamin C along with quercetin has synergistic effect in treating COVID-19 patients	[93,99,100,101]
4.	Apigenin	Acts against the African swine fever virus by decreasing its protein synthesis In picrona virus it inhibits the viral protein entry In enterovirus-71 it inhibits the viral RNA association with transacting factors In HCV it inhibits the viral replication	[103,104,105,106,202]
5.	Baicalin	In case of HBV, it inhibits the template for viral protein and DNA synthesis In the case of HCV also it inhibits the protein and RNA synthesis The replication of the avian influenza virus is inhibited by interfering with the neuraminidase activity In influenza A virus infection it stimulates the production of IFN-γ in the CD^4+^ and CD^8+^ cells It was found to have increased binding property with NS3/NS2B protein and also has closer interaction with NS5 protein of the dengue virus	[108,109,110,112,120]
6.	Luteolin	In HIV it inhibits the clade B and C–T at driven transactivation In Epstein-Barr virus it decreases the activity of early genes *Rta* and *Zta* by deregulating the binding of the transcription factor Sp1 In enterovirus 71 and coxsackievirus A 16 it disrupts the viral replication Luteolin inhibits the viral entry and fusion of SARS CoV-2 with human receptors	[113,114,115,116,117,118,119,120,210]
7.	Rhamnose residue containing kaempferol	In HIV, it inhibits the reverse transcriptase enzyme In H1N1 and H9N2, it affects the neuraminidase activity In Japanese encephalitis virus, it inhibits the RNA frame shift Inhibits coronavirus release by effecting 3a channel Inhibits N3 binding site in the SARS-CoV-2 M^pro^	[122,124,125,126]
8.	Kaempferol 3,7-bisrhamnoside	Effective against HCV NS3 protease	[123]
9.	Triterpene saponin	It acts against the HSV-1 and poliovirus 2 by protecting the host cells from cell damage and also by decreasing the viral production	[131]
10.	Triterpenoid saponin TS21	In HSV it inhibits the viral capsid protein synthesis and also replication Saponins has anti-inflammatory activities, anti-proliferative effect, immunomodulatory and antiviral activities including SARS-CoV	[134,135,136]
11.	Niranthin	In HBV infection it inhibits the antigen expression In the case of duck HBV infection it inhibits the DNA replication	[139,140]
12.	Nordihydroguairetic acid	In the case of HCV, it affects the viral proliferation by inhibiting the genome replication and viral assembly It suppresses the influenza A virus replication	[141,142,143,144]
13.	Terameprocol	It inhibits the West Nile virus replication	[145,146,147,148,149]
14.	Arctigenin	It inhibits the expression of P17 and P24 proteins of the HIV In the case of HIV-1 and HSV, it protects the host by increasing the production of IFN	[150,151,152,153,154]
15.	Yatein	In HeLa cells, it inhibits the HSV-1 virus DNA synthesis by inhibiting the expression of ICP0 and ICP4	[155,156]
16.	Diphyllin	It inhibits the vacuolar ATPase in case of zika virus In the case of influenza A virus, it inhibits the infection by interfering with the downstream process	[157,158,159,160,161]
17.	Patentiflorin A	Prevents the fusion of the cell membrane of the host by inhibiting the acidification of endosomal and lysosomal cells In HIV it inhibits the reverse transcriptase	[160,161,162,163,164]
18.	Clemastanin B	In influenza A virus it affects the viral endocytosis and also the ribonucleoprotein export	[164,165,166,167]
19.	Silymarin C	It inhibits the HCV production and also increases the anti-inflamatory and antiproliferative gene expression Lignins exhibits antiviral property by inhibition of viral replication, protein and cytokine expression, and apoptosis thus they may have effect on SARS-CoV-2	[168,169,170,171]
